# Mitochondrial Dysfunction in the Cardiovascular Disease Continuum: Problems of Studying the Progression During the Follow-Up of the Pathologies

**DOI:** 10.3390/ijms262110497

**Published:** 2025-10-29

**Authors:** Ganna Nevoit, Maksim Potyazhenko, Ozar Mintser, Gediminas Jarusevicius, Alfonsas Vainoras

**Affiliations:** 1 Laboratory for Automatization of Cardiovascular Investigations, Cardiology Institute, Lithuanian University of Health Sciences, LT-44307 Kaunas, Lithuania; 2 Department of Internal Medicine and Emergency Medicine, University Poltava State Medical, 36011 Poltava, Ukraine; 3 Department of Fundamental Disciplines and Informatics, Shupyk National Healthcare University of Ukraine, 04112 Kyiv, Ukraine

**Keywords:** cardiovascular diseases, cardiovascular continuum, mitochondrial dysfunction

## Abstract

This perspective piece extrapolates knowledge of mitochondriology to the clinical aspects of cardiovascular disease (CVDs) development. The aim was to deepen the understanding of the etiopathogenesis of CVDs by conceptualizing the systemic involvement of mitochondrial dysfunction mechanisms in their follow-up. A theoretical comparison of mitochondrial status and mitochondrial dysfunction across stages of the cardiovascular continuum was performed based on a systematic analysis of the scientific literature data using general scientific, theoretical, and logical methods and normative rules. Conceptual aspects of the involvement of mitochondrial dysfunction (MD) mechanisms at each stage of the CVDs continuum were identified. MD is a dynamic, complex, multifactorial process that is characterized by quantitative and qualitative changes in the mitochondrial pool of human body cells during the development of CVDs. MD is a fundamental participant in the pathogenesis of CVDs, predetermining the nature and features of the clinical manifestation and course of the disease in each patient. MD has distinctive features at each stage of the catamnesis of CVDs and can be classified according to this principle. The development of objective methods for assessing the degree of MD and its classification criteria is a promising task for future scientific research.

## 1. Introduction

The cardiovascular disease (CVDs) are a significant medical and social problem that requires a solution. CVDs have the highest prevalence and mortality rates and occupy a leading place among chronic non-communicable diseases (NCDs) among the entire global population [1,2,3]. Every year, CVDs cause the death of about 17.9 million people. Heart attacks and strokes are the cause of death from CVDs in four out of five deaths. A threatening aspect is that a third of these deaths occur prematurely in people under 70 years of age [4]. It is due to the contribution of CVDs that NCDs have now reached the level of a pandemic [4,5,6,7,8]. CVDs cause severe economic losses and are an obstacle to achieving the 2030 Sustainable Development Goals [9,10]. The World Health Organization (WHO) has been paying primary attention to the prevention and treatment of CVDs for several decades [9,11,12,13,14,15,16].

Thus, CVDs continue to be a challenge for modern scientists. Therefore, the search for new ways to solve the problem of CVDs is relevant for modern medical science. In the last few decades, a fundamental breakthrough in understanding the underlying mechanisms of the pathogenesis of CVDs and NCDs has been outlined. This is associated with significant achievements in molecular biology in studying the role of mitochondria in cell functioning in normal and pathological conditions [17,18]. It has become clear that the mitochondrial dysfunction (MD) is one of the key pathogenetic mechanisms of the occurrence and progression of CVDs and NCDs [19,20,21]. Today, it is clear that mitochondria are key organelles of the cell, which provide its bioenergetics, metabolic processes [22], and control vital activity and death [23]. This has become a serious foundation for further transdisciplinary extrapolation of mitochondriology knowledge into clinical medicine. This poses new challenges for scientists to identify additional connections and mutual influences between mitochondrial dysfunction in the cells of different tissues across various organs. It is now crucial to formulate systematic theoretical comparisons of the morphological state of mitochondria, the degree of MD, and the clinical manifestations of CVDs and other types of NCDs. For further medical progress in this area, it is advisable to understand how exactly the degree of MD arises and changes in the process of CVDs development, in which tissues of organs and in what sequence this occurs, and what the objective influence on this change is such as nutrition, physical activity, various types of pharmacological therapy, etc. Therefore, the aim of this perspective piece was to become a theoretical starting point for deepening the knowledge of the etiopathogenesis of CVDs by creating a working concept of clinical ideas about how the mechanisms of MD fit into the catamnesis of CVDs. The tasks were set based on the conducted system analysis to compare the existing knowledge of mitochondriology with modern clinical concepts of the stages of the clinical development of CVDs.

**Figure 1:**
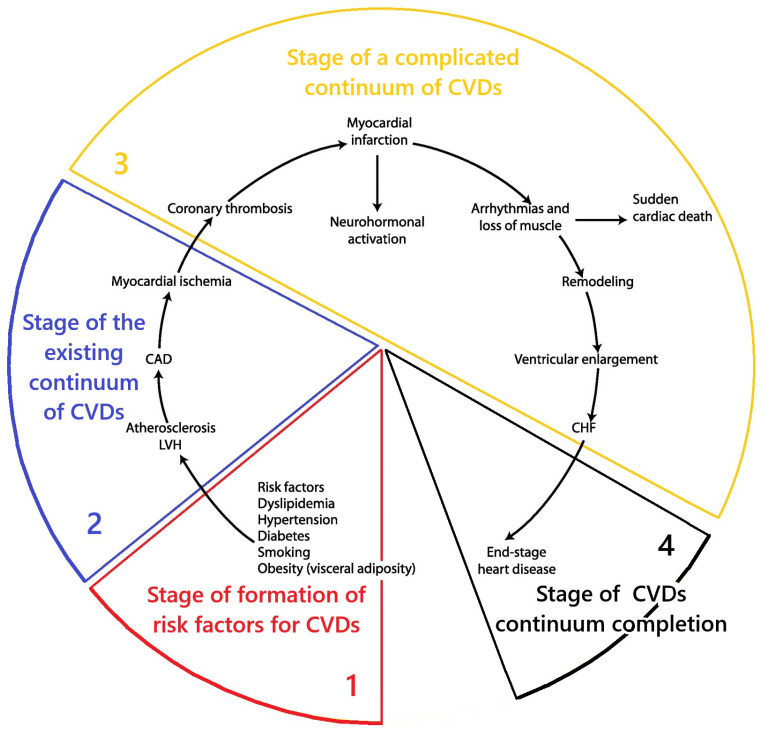
Scheme of the stages CVDs continuum. Fragment [25] used. Note: CAD—coronary artery disease; LVH—left ventricular hypertrophy; CHF—heart failure.

The basis of modern concepts of CVDs catamnesis is the theory of the CVDs continuum. These are generally accepted theoretical concepts that describe the dynamics of CVD pathology development. According to this theory, CVDs exhibit a regular sequence of pathological changes that gradually develop over time and complement the existing preliminary pathological disorders. These ideas were first formulated and substantiated in the works of V. Dzau and E. Braunwald [24,25,26] (Figure 1).

According to the CVDs continuum theory, pathology does not occur immediately. It is preceded by the first initial stage, during which pathogenetic factors affect the body. This gradually leads to metabolic disorders in the body, which in turn become the basis for the development of pathology. This time interval of pathogenesis is referred to as the “CVDs risk factor formation stage”. The further influence of pathogenic mechanisms ultimately leads to the development of clinical manifestations of the disease. This period is referred to as the “stage of the existing CVDs continuum”. Further progression of the pathology leads to the development of complications in the form of myocardial infarction, stroke, cardiac arrhythmia, etc. This is the “stage of complicated CVDs continuum”. Further progression of the disease leads to the “stage of completion of the CVDs continuum”—to the death of the patient from CVDs. Therefore, in this perspective piece, each section provides a brief description of the role of MD in the pathogenesis of CVDs at each of these stages of the CVDs continuum model [24,25,26]. To this end, studies have been conducted in the scientific literature aimed at extrapolating knowledge about mitochondrial dysfunction to the stages of development of the cardiovascular disease continuum [24,25,26]. This approach is justified by the need to link changes in mitochondrial morphology and function with the existing scientific model of the clinical progression of CVDs to deepen our understanding of their etiopathogenesis. General scientific methods of theoretical research were used: separation and integration of parts of the system under study, logical research, mental analysis, deduction, induction, and knowledge synthesis.

## 2. MD and Cardiovascular Continuum: Results of System Analysis

In this perspective piece, the results of the performed theoretical study are presented in the form of the corresponding subsections:

- Conceptualization of the key aspects of MD at the stage of functional health and formation of risk factors of the cardiovascular continuum (CVDs risk factor formation stage);
- Conceptualization of the key aspects of MD at the stage of the existing cardiovascular continuum;
- Conceptualization of the key aspects of MD at the stage of complicated cardiovascular continuum (stage of complicated CVDs continuum);
- Conceptualization of the key aspects of MD at the stage of the end of the cardiovascular continuum (stage of completion of the CVDs continuum).

### 2.1. Conceptualization of Key Aspects of MD at the Stage of Functional Health and Formation of Risk Factors of the Cardiovascular Continuum

**Figure 2:**
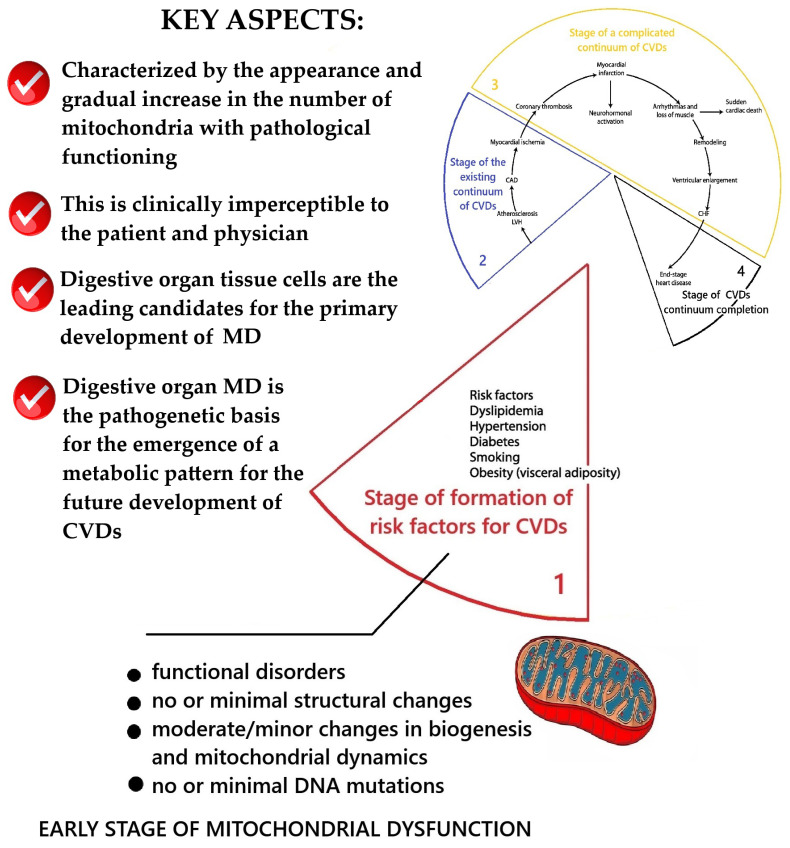
Scheme of the key aspects of MD at the stage of functional health and formation of risk factors of the CVDs continuum. Fragment [25] used. Note: CAD—coronary artery disease; LVH—left ventricular hypertrophy; CHF—heart failure.

Based on the system analysis, it was established that the stage of formation of risk factors of the cardiovascular continuum is directly related to the initial stages of the formation of MD. The results of the conceptualization of key aspects of MD at this stage are presented in Figure 2.

A fundamentally new conclusion is the conceptualization that mitochondrial dysfunction should arise primarily in the tissue cells of the gastrointestinal tract. The logic of this idea was based on the argument that the emergence of the so-called metabolic pattern (hypercholesterolemia, dyslipidemia, insulin resistance, etc.) and typical pathological changes in tissues (endothelial dysfunction, systemic inflammation, atherosclerosis) requires the emergence of pathological abnormalities in the liver and other tissues of the gastrointestinal tract. Based on this, the working concept of the emergence of MD at the stage of functional health and the formation of risk factors for the cardiovascular continuum, as presented below, was developed.

Below is a working concept of the emergence of mitochondrial dysfunction at the stage of functional health and the formation of risk factors of the cardiovascular continuum.

Indeed, as practical clinical experience demonstrates, in most cases, functionally healthy people already in their childhood, under the influence of factors of unhealthy diet, physical inactivity, and stress (e.g., schooling, etc.), begin to develop vegetative dysfunction and functional diseases of the upper and lower gastrointestinal tract (dyskinesia of the digestive organs) [27,28,29,30,31,32]. A study of comorbidity in accordance with the stage of occurrence of cardiovascular lesions confirmed the primary appearance of disorders of the gastrointestinal tract at the stage of risk factor formation and revealed that pathology of the gastrointestinal tract was present in 100% of cases in all patients with CVDs [33]. Functional pathology of the digestive organs creates conditions for the gradual emergence of a metabolic pattern. The mechanisms of MD development under the influence of risk factors have been described in detail in reviews [17,19,34]. The key risk factor among those contributing to the pathogenesis of MD at this stage is quantitative and qualitative nutritional disorders. The central pathogenic component is systematic overeating, accompanied by the absence of sufficient periods of hunger (over 12 h per day). A large number of food substrates constantly entering in excess serves as a metabolic load for mitochondria, triggers a cascade of molecular mechanisms for the development of MD, and disrupts mitochondrial biogenesis [19,35]. Mitochondria must process all food substrates that have entered them. Therefore, with their excess, the nature of metabolic processes is transformed, and an excessive generation of hydrogen protons occurs. Thus, the mitochondria try to remove excess energy substrates. In this case, a pathological change in the electromagnetic potential on the mitochondrial membranes may occur. Changes in the course of electromagnetic processes in mitochondrial membranes negatively affect their metabolic and synthetic activity, promote the occurrence of pathological signaling, and increase the synthesis of free oxygen forms (ROS) [19,36]. All of these trigger further pathological molecular mechanisms. Of great importance are the pathological activation of lipids, proteins, and nucleic acid peroxidation and disruption of the signal interaction between molecules in cells [19,37,38]. The result of this, respectively, is, on the one hand, mutations in mitochondrial DNA [39,40,41] and, on the other hand, disruptions in the Krebs cycle and oxidative phosphorylation [42,43,44].

On the other hand, this leads to the generation of proinflammatory signals by mitochondria, initiating degradation mechanisms in the cell (premature aging, apoptosis, etc.) [45,46]. These pathological deviations lead to one result: a decrease in the ability of mitochondria to produce energy substrates [19]. Thus, a completely logical paradox arises: a constant supply of excess energy in the form of food substrates does not give cells more energy, but, on the contrary, leads to a gradual emergence of cellular energy deficiency due to the emergence of MD. At the same time, pathological conditions for the internal existence of the cell gradually begin to arise inside the cell in the form of a change in its electromagnetic parameters and function. Over time, this will clinically manifest as a disruption of the functions of the tissues in organs where MD occurs in their cells, which will mark the transition to the stage of an existing disease.

An essential aspect for the future development of CVDs is the disruption of mitochondrial biogenesis caused by the absence of extended periods (about 12 h) of daily hunger [19,35,47]. Constant regular consumption of food by humans, especially eating in the evening and at night, creates conditions for a continuous influx of food substrates to the mitochondria and turns off the mechanisms of their fusion into the mitochondrial reticulum [48,49,50]. This leads to the fact that mitochondria constantly exist in a fragmented state, and the mechanisms of “natural selection” by the death of defective mitochondria stop working. This contributes to the gradual formation of a pool of faulty mitochondria in the cell. The existence of defective mitochondria and an increase in their number in the cell is associated with an increase in pathological mitochondrial signals in the cell and the initiation of pathogenetic mechanisms [19,51]. Over time, this will lead to a qualitative change in the cell’s condition, to its pathology, and will also mark the transition from a state of health to the stage of an existing disease. Disturbances in the qualitative composition of food supplement the adverse effects of systematic overeating. A systematic shortage in the diet of vitamins [51,52,53] and microelements [54,55,56,57,58,59,60,61,62,63,64] necessary for the functioning of mitochondrial enzyme chains leads to a decrease in their activity and reduces the production of energy substrates by mitochondria. Deficiencies of macronutrients (for example, phospholipids, individual amino acids) disrupt the processes of remodeling the membrane structures of mitochondria [65,66,67,68,69]. This leads to the emergence of morphological defects of membranes. The consequence of this is a decrease in their functional activity, which disrupts their ability to fuse into the mitochondrial reticulum and can lead to their premature destruction [19]. Similar adverse effects on mitochondria are caused by the excessive intake of specific nutrients with food. From childhood, the nature of modern human nutrition in our society contributes to the intake of large amounts of preservatives, stabilizers, and food additives [70,71], excess table salt [72], and other chemical agents that are unnatural for the human body [73,74,75]. A significant contribution to this can be made by human life in conditions of anthropogenic pollution of the environment [76]. This causes an additional load on the membrane components of mitochondria with potentially toxic substances that come from water, air, contact with human skin, etc. These create an external toxic load and lead to damage, blocking mitochondrial enzyme chains, and MD [19].

Logically, the first target of the adverse effects of nutritional disorders is mitochondria in the cells of the gastrointestinal tract and liver. The primary disruption of mitochondrial functions should occur in them, leading to the gradual formation of a pathological circle of comorbid pathology from the digestive organs, dyslipidemia, hypercholesterolemia, and so on.

Certainly, pathological changes in the digestive organs that precede the development of CVDs have multifactorial etiological causes. It is generally accepted that the gastrointestinal tract is under the constant influence of the external environment in the form of food and viral and bacterial agents entering it. Thus, the adverse effect of pathogenic microflora on mitochondrial function has been proven. In particular, Helicobacter pylori causes the fragmentation of mitochondria with the release of cytochrome C into the cytosol, mitochondrial initiation of apoptosis [77,78]. Theoretically, other pathogenic and opportunistic microbial agents that enter the upper gastrointestinal tract with food can also have an adverse effect. This is an important direction for future research. It is essential to note that the gastrointestinal tract organs are quite energy-dependent due to the high functional activity of their tissues. Therefore, the occurrence of chronic energy deficiency due to MD will undoubtedly affect their functional state and gradually lead to the appearance of pathomorphological changes. For example, the mucous membranes of the stomach and duodenum take an active part in the processes of digestion, immunological and endocrine responses, have a high regenerative potential, and a close neuroendocrine connection with the pancreas, liver, and gall bladder [79,80]. This explains their high energy needs, active level of metabolism, and functional interdependence. Under the influence of such pathogenetic factors as irrational, inadequate nutrition, including chemicalized products with prooxidant properties, gradual damage to the mitochondria of cells occurs [19], and MD becomes a component of the pathology of the organs of the upper gastrointestinal tract. Using mitochondrial diseases as an example, it has been established that MD is clinically manifested in gastrointestinal tract lesions by poor appetite, dysfunction of the gastroesophageal sphincter, constipation, dysphagia, gastroparesis as well as in pancreatitis and hepatopathy—vomiting, pseudo-obstruction of the gastrointestinal tract [81,82,83,84]. A logical explanation for the occurrence of various types of gastrointestinal motility disorders can be attributed to altered smooth muscle cell functions and autonomic dysfunction, characterized by decreased parasympathetic tone resulting from mitochondrial dysfunction and the associated metabolic disorders. The maximum manifestations of mitochondrial dysfunction are pronounced clinical and morphological changes in tissues and organs. For example, dry mouth, periodontal disease, tracheoesophageal fistula, duodenal stenosis, atresia or imperforate anus, liver cysts, pancreatic lipomatosis, cysts, congenital stenosis or obstruction of the gastrointestinal tract, recurrent intestinal perforations, diverticulosis, or pneumatosis intestinalis [83,84,85]. As a rule, the basis of such significant clinical manifestations is MD, caused by a genetic disorder in mitochondrial nucleic acids [85,86,87,88]. This is not typical for the initial scenarios of CVDs and NCDs follow-up, except for primary mitochondrial diseases. However, given the significant polymorbidity and comorbidity of modern patients with CVDs and NCDs, it can be assumed that acquired secondary gene disorders of the mitochondrial genome also contribute to the comorbidity and multisystem disorders in CVDs and NCDs at the stages of clinical manifestations and the occurrence of complications. From the standpoint of systems medicine, substantiating the role of upper gastrointestinal tract pathology in the formation of CVDs risk factors, it should be noted that in terms of improving the issues of the etiopathogenesis of NCDs, the liver should be considered not only as a metabolic, but also as an immune center of the human body [89]. The liver is a site of complex immunological activity, participates in the production of acute phase proteins, coagulation factors, and complement, cytokines, and albumin, and contains a large population of resident immune cells [89,90].

Innate lymphocytes in the liver include natural killer cells, natural killer T cells, mucosa-associated invariant T cells, and γδ T cells [91,92]. The liver contains many cells of the adaptive immune system: CD8+ T cells, activated T cells, and memory T cells [89,93]. Hepatocytes express varying levels of primary histocompatibility complex class II molecules and are capable of presenting antigens to classical T cells [94]. The healthy liver maintains basal levels of cytokines including the expression of pro-inflammatory interleukins IL-2, IL-7, IL-12, IL-15, and IFNγ as well as anti-inflammatory interleukins IL-10, IL-13, and transforming growth factor β [95,96]. Moreover, liver mitochondria are organelles where not only metabolic pathways converge, but they are also capable of altering innate and adaptive immune responses, releasing immunostimulatory molecules, modulating the microenvironment, and ensuring immunometabolic processes as well as serving as sites of transmission for inflammatory signals [97,98].

Taking the above into account, from the standpoint of systems medicine, the role of the liver in the formation of factors for the development of CVDs and NCDs can be described as follows. Factors of non-compliance with a healthy lifestyle, autonomic dysfunction, and pathological signaling from other organs of the gastrointestinal tract form the basis of constant excessive stress effects on hepatocytes and lead to the appearance of mitochondrial dysfunction in the cells of organs (primarily the gastrointestinal tract, in particular the liver). The degree and mechanism of MD may differ and change depending on the combined effect of individual etiological factors and the available adaptive reserves of the patient’s body. Thus, MD has been proven in various pathological conditions of the liver [97,98,99,100,101,102,103,104,105]. Under the influence of unhealthy lifestyle factors, MD arising in hepatocytes changes the functioning of the liver. In hepatocytes, the hyperproduction of free fatty acids begins to appear due to impaired beta-oxidation in the mitochondria, dyslipidemia, and hyperactivation of free radical oxidation [19,34]. Hyperproduction and oxidation of free fatty acids in mitochondria contribute to the formation of free radicals, which gradually deplete the antioxidant defense system of hepatocytes, initiating their further damage. Disorders of lipid homeostasis of hepatocytes gradually lead to the development of fatty liver disease. Synthetic activity of hepatocytes altered due to MD leads to the clinical appearance of disorders of the organism’s lipid homeostasis—dyslipidemia and hypercholesterolemia. Low-density lipoprotein cholesterol and cholesterol accumulated in endothelial cells are modified by lipid peroxidation, which contributes to the appearance of damage to the mitochondria of endothelial cells, creating an energy-deficient state in them. A decrease in the transmembrane potential of mitochondria and an increase in the mitochondrial production of reactive oxygen species (ROS) trigger pathways of endothelial cell apoptosis through the activation of mitochondrial complex II and nicotinamide adenine dinucleotide phosphate oxidase and inflammation [106,107,108,109]. Dyslipidemia and endothelial dysfunction become key chains in the occurrence and progression of atherosclerosis, and accordingly, contribute to the appearance over time of clinical manifestations of coronary heart disease and hypertension [19], which will correspond to the transition to the next stage of the continuum—existing CVDs.

As already noted, clinically mitochondrial dysfunction of hepatocytes can manifest itself as the appearance of deviations in biochemical parameters of the liver of varying degrees, changes in the physicochemical parameters of bile, and subsequently the occurrence and manifestation of chronic diffuse liver disease and biliary tract pathology [97,98,99,100,101,102,103,104,105]. Thus, from the standpoint of systems medicine, it is essential to understand that one of the key roles in the etiopathogenesis of CVDs and NCDs is played by pathological changes in liver function. This is because it is the MD of hepatocytes through a pathological increase in the production of ROS and reactive nitrogen species, a decrease in β-oxidation with corresponding immune and metabolic disorders, and bioenergetic insufficiency that gradually leads to the emergence of a metabolic pattern and the gradual appearance of such systemic pathological conditions as endothelial dysfunction and atherosclerosis.

In the intestine, with an unbalanced diet, a microbial imbalance is formed [110,111], which, due to the connection between the microbiome and the mitochondrial biome, also contributes to the development of MD [112,113,114,115,116,117,118,119,120,121]. Chemicalized food components can have a direct damaging effect on the membranes and mitochondria of intestinal cells, also causing MD. This gradually creates conditions for the development of pathological conditions in the lower gastrointestinal tract including intestinal microbiome disorders as well as functional, inflammatory, and degenerative changes in the intestinal tract. All of the above pathological deviations, as basic pathogenetic links, create a negative epigenetic background for the health of the entire organism, forming pathological circles with the subsequent involvement of the corresponding functionally related organs of the gastrointestinal tract. Further imbalance of the symbiotic system “mitobiota and microbiota” with chronicization of the complex deficiency of functional ingredients against the background of stress effects, chronic energy-cytodeficiency, bioenergetic hypoxia, and pathological signaling from mitochondria in the cells of the gastrointestinal tract will lead to the progression of their membranes. In a greater number of cells, disorders of communication between cells in tissues, acceleration of cellular aging, defects in the processes of cell proliferation and apoptosis, and chronic inflammation [122,123,124,125,126,127,128]. This will support the gradual progression and aggravation of pathological changes in the preclinical stage of NCDs. At the subsequent stage of the NCDs continuum, it will lead to comorbidity.

Physical inactivity as a risk factor for CVDs does not have a direct damaging effect on mitochondria. However, chronic physical inactivity provides an additional impetus for degenerative changes in mitochondria, a decrease in their number in muscles, and muscle hypertrophy. It creates a chronic hyperergic state in the human body [129]. Functionally, muscles are the body’s bioenergetic station, which generates electromagnetic and other types of energy for the entire body, thereby ensuring the completion of the cycle of food processing and assimilation [130]. In this case, excess food, not converted into energy in the muscles, begins to accumulate in regional fat depots, changing body composition and causing obesity [131,132]. In functionally healthy young individuals who do not regularly engage in sports, during this period, changes in body composition occur at the organism level, characterized by an increase in the percentage of fat (pre-obesity, obesity, visceral obesity) and a deficit in the percentage of muscle tissue, as indicated by impedance meter data. Therefore, muscle mass deficit is proposed to be considered as a new early predictor of the occurrence of CVDs and NCDs [133]. Additionally, physical inactivity triggers the hypothalamic pathway, leading to the occurrence of vegetative dysfunction in the cardiovascular system and throughout the body, and maintains a general energy deficit and a state of bioenergetic tissue hypoxia at the organismal level [19,134,135].

The cardiovascular system is one of the most energy-dependent in the human body. Thus, the heart muscle alone processes about 10% of all adenosine triphosphate (ATP) formed by the body per day (on average 6 kg out of 65 kg) [136]. Of course, the systemic hypoergic state and tissue bioenergetic hypoxia should inversely affect its functioning. The fact of significant energy dependence of the cardiovascular system and MD of cardiomyocytes was established thanks to the study of the characteristics of their damage in mitochondrial diseases [137,138,139]. It has now been proven that mitochondrial physiology and mitochondrial biogenesis play a key role in the initiation and progression of CVDs caused by oxidative damage [140,141,142]. In cardiomyocytes and endothelial cells, it is the mitochondria that play the main role in ensuring their normal functioning: they produce ATP, regulate the work of such mediators of cellular signaling as calcium and ROS, interact with other organelles, and make a significant contribution to endothelial dysfunction [143,144,145]. ROS in low concentrations are important for the implementation of vascular signaling processes and regulate the activity of protein mediators, enzymes, and ion channels in endothelial cells [146,147].

As a summary, it can be noted that the factors of an unhealthy lifestyle and the risk of developing atherosclerosis and ischemic heart disease, due to the mechanisms of mitochondrial dysfunction, lead to an increase in the levels of ROS and nitrogen production [35,147,148,149]. ROS and active nitrogen species play an important role in the occurrence of the main pathogenetic links of CVDs—endothelial dysfunction and atherogenesis, since they are involved in processes such as the dysfunction and apoptosis of endothelial cells, activation of matrix metalloproteinases, growth of vascular smooth muscle acids, and their migration, and low-density lipoproteins [150,151,152]. Excessive production of ROS and nitrogen contributes to the occurrence of inflammatory vascular reactions, atherosclerotic vascular lesions, and causes the further progression of atherosclerosis. With endothelial dysfunction, the production of nitric oxide by endothelial NO synthase decreases. Together with the overproduction of ROS, this continues to initiate the development of atherosclerosis. Excess ROS leads to the formation of peroxynitrite anion, which in turn inhibits tetrahydrobiopterin, an essential cofactor of endothelial NO synthase, leading to a further decrease in NO synthesis. A significant decrease in NO levels also promotes the opening of mitochondrial ATP-dependent K channels, which entails the release of ROS, and again, the stimulation of atherogenesis [153,154,155]. Oxidative damage to mitochondria has been shown to cause endothelial dysfunction in experimental studies [156,157]. Clinical studies have also shown a link between mitochondrial dysfunction and endothelial dysfunction in patients with coronary heart disease [158,159,160]. Thus, ROS production during mitochondrial dysfunction is a key mechanism by which mitochondria are involved in the development of CVDs such as coronary heart disease, cardiomyopathy, ischemia–reperfusion injury, heart failure, and arrhythmia. ROS also induce direct damage to nucleic acids: single- and double-strand breaks, deletions, and chromosomal translocations, which contribute to both genomic and mitochondrial instability [161,162,163,164]. This may be a contributing factor to an individual scenario involving the development of comorbid oncological pathology, potentially leading to the further progression of the general continuum of NCDs.

Alcohol consumption and smoking can significantly increase the risk of early atherosclerosis by negatively affecting mitochondrial function [164,165,166,167,168]. In addition to endothelial damage, platelet activation, oxidation of low-density lipoprotein cholesterol, and the atherogenic effects of smoking and alcohol can also be due to oxidative damage to mitochondrial DNA, the appearance of deletions in it, and the loss of mitochondrial membrane potential [169].

### 2.2. Conceptualization of the Key Aspects of MD at the Stage of the Existing Cardiovascular Continuum

**Figure 3:**
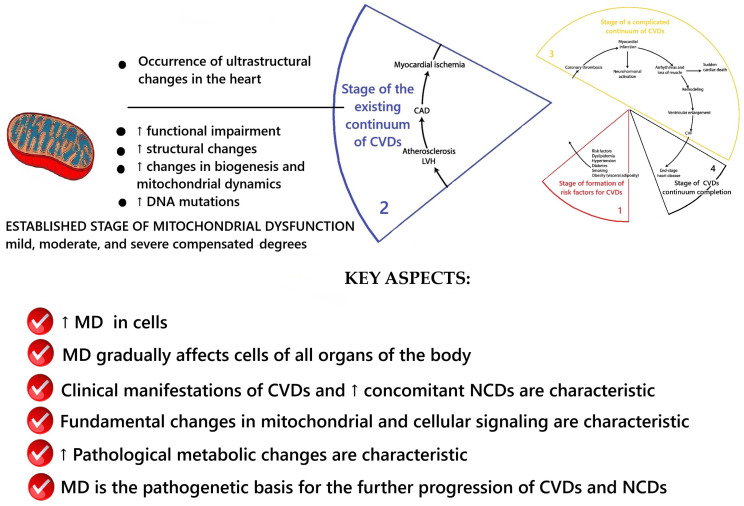
Scheme of the key aspects of MD at the stage of the existing CVDs continuum. Fragment [25] used. Note: CAD—coronary artery disease; LVH—left ventricular hypertrophy; CHF—heart failure.

According to V. Dzau and E. Braunwald [24,25,26], the stage of the existing cardiovascular continuum is characterized by the appearance of clinical manifestations of CVD: primarily coronary heart disease and arterial hypertension. The results of the conceptualization of key aspects of MD at this stage are presented in Figure 3.

At this stage, the processes of the progression of atherosclerosis and endothelial dysfunction, pathological disorders between organs and organ systems in the body, continue to occur. This leads to a gradual increase in comorbidity. It has been confirmed that it is at this stage of follow-up in patients with CVDs that comorbidity increases significantly. During the period of complications, it begins to reach its maximum, and almost all organ systems of the body are involved in the pathological processes of NCDs. For example, in one study [33], during a retrospective analysis of comorbid pathology in a group of 69 patients with CVDs who had no history of cardiovascular complications, the presence of concomitant pathology of the gastrointestinal tract (100%), musculoskeletal system (36%), sensory organs and nervous system (100%), urinary (36%) and endocrine system (6%) was established; the CIRS index was 8.55 ± 2.66 and the SCORE index was 11.4 ± 3.4%. Retrospective analysis of comorbid pathology in 72 patients with CVDs who had a history of myocardial infarction revealed the presence of concomitant pathology of the gastrointestinal tract (100%), musculoskeletal system (72%), sensory organs and nervous system (100%), urinary (86%), and endocrine system (14%); the CIRS index was 13.51 ± 3.84 and the SCORE index was 18.4 ± 6.2%. There was a significant increase in the average diagnostic accuracy in subgroups with a predominantly complicated cardiovascular continuum (*p* < 0.05), indicating an increase in comorbidity with further progression of CVDs compared with their complications [33]. An explanation was found for this, which is that at the stage of existing CVDs, MD remains the most critical integral pathogenetic mechanism, leading to the further development of cardiovascular system pathology and other organ system pathologies in the human body. It is the continuing increase in MD that theoretically explains the continuing progression of atherosclerosis under the influence of the main risk factors for CVDs and the further development and aggravation of manifestations of comorbidity [19,34].

At the stage of the existing cardiovascular continuum, an unhealthy lifestyle with physical inactivity, poor nutrition, chronic exotoxic nutrient load, and stress continues to support the pathological “metabolic circles” formed at the stage of formation of the cardiovascular continuum. It maintains elevated levels of ROS production [170]. This continues to support the main pathogenetic links of CVDs—endothelial dysfunction, systemic chronic inflammation, atherogenesis, etc. This causes the involvement of an increasing number of endothelial cells in different parts of the body in pathological processes. This is accompanied by dysfunction and apoptosis of endothelial cells, activation of matrix metalloproteinases, an increase in vascular smooth muscle cells and their migration to the intima, expression of adhesion molecules, and oxidation of low-density lipoproteins [171,172,173,174,175,176]. It has been established that the atherogenic process is accompanied by structural and functional disturbances in the mitochondria of target cells and in the endothelial cells of atherosclerotic plaques [34,177,178]. Excessive production of ROS and nitrogen continues to cause inflammatory vascular reactions, maintaining the cycle of progression of atherosclerotic vascular lesions [178,179] and contributes to the further progression of CVDs, and accordingly, the cardiovascular continuum. It has been proven that the load of macrophages with free cholesterol, characteristic of CVDs, is also associated with mitochondrial dysfunction [180,181,182]. The pathogenetic significance of oxidized lipoproteins at the stage of existing CVDs is that they continue to cause apoptosis in all cells involved in atherogenesis: endothelial and smooth muscle cells, macrophages, and T-lymphocytes [183].

Like atherosclerosis, the development and progression of arterial hypertension is also pathogenetically determined by unhealthy diets (excessive salt consumption, a diet high in saturated fat and trans fats, low intake of fruits and vegetables), physical inactivity, consumption of tobacco and alcohol, being overweight or obese [184], endothelial dysfunction, and oxidative stress [185,186,187,188,189,190,191,192,193,194], respectively. It has been proven that arterial hypertension is accompanied by a disruption of mitochondrial ATP synthase in cardiomyocytes, and they have a mitochondrial calcium load [148,195,196] as well as existing changes in the mitochondrial genome [197,198,199]. Mitochondrial dysfunction in the endothelial and smooth muscle cells of blood vessels is associated with hypertension [192]. Upregulation of dynamics-related protein 1 (DRP1) and downregulation of optic atrophy-associated protein 1 (OPA1) impairs mitochondrial dynamics in the small mesenteric arteries of SHR. This results in increased release of mitochondrial reactive oxygen species, inflammation, and growth factor signaling, leading to media thickening and luminal narrowing [200]. Mitochondrial dysfunction in vascular smooth muscle cells stimulates proliferation and a phenotype switch from a contractile to a proliferative state. This leads to vascular remodeling and increased vascular stiffness [201,202]. Reduced mitochondrial fission may inhibit vascular smooth muscle cell migration by altering mitochondrial bioenergetics and ROS levels [203]. Mitochondria in endothelial cells control endothelial function and ROS generation in them [204], influence endothelial pathophysiology through Ca^2+^ signaling [205], nitric oxide production [190], apoptosis, and autophagy [206]. Therefore, mitochondrial dysfunction of endothelial cells contributes to end-organ damage and the formation of comorbidities in CVDs. Hyperacetylation of mitochondrial proteins (SIRT3, SOD2, or CyPD) impairs mitochondrial metabolism and causes oxidative stress and hypertensive vascular dysfunction [190].

The fundamental difference of this stage is that significant changes occur in the mitochondria of cardiomyocytes. This causes pathomorphological processes of myocardial remodeling and further pathogenesis of CVDs, with the subsequent development of heart failure and the occurrence of arrhythmias [206]. There is an increasing shift in the balance toward mitochondria being in a defragmented state, a decrease in the area of mitochondria, a reduction in the density of mitochondrial cristae, and a violation of the distribution of mitochondria in cardiomyocytes [206]. In the early stages of pathological cardiac hypertrophy, fragmented mitochondria are located in clusters within neighboring sarcomeres [207]. With the loss of mitochondrial cristae and the swelling of mitochondria, the stage of vacuolar degeneration of mitochondria occurs. This will lead to their rupture at later stages of CVD development [208,209]. Conditions for calcium overload of cardiomyocyte mitochondria gradually arise and increase [195,210]. This is especially characteristic of cells that enter the apoptotic process [208,211]. This fact has been confirmed in patients with coronary heart disease with heart failure and is more pronounced in patients with a decrease in cardiac ejection fraction and aortic stenosis [196,209]. With the progression of CVDs, multiple lysosomes appear in cardiomyocytes as a sign of increased mitophagy [196,209]. At the early stages of CVDs, this can be regarded as a cardioprotective process that ensures mitochondrial quality control. However, during the increased activation of mitochondrial dysfunction signaling pathways, apoptosis and the progression of cardiac remodeling have been associated with pathological excessive activation of mitophagy [212,213]. This is confirmed by the study of molecular markers of autophagy and mitophagy in patients at different stages of heart failure [12,196]. More pronounced lysosome dysfunction due to the lipid peroxidation of lysosomal membranes and accumulation of lipofuscin material in lysosomal bodies has been established in biopsy materials of patients with III–IV classes of heart failure according to the New York Heart Association (NYHA) classification [196,209].

With the progression of CVDs, changes in the expression of the post-translational modification of mitochondrial dynamic proteins in myocardiocytes have been established. This has a fundamentally pathogenic character and indicates profound, underlying pathological changes in the life and functioning of mitochondria at the stage of the existing cardiovascular continuum. For example, the expression of MFN1 and MFN2 is increased in patients with ischemic heart disease, with non-ischemic cardiomyopathy [214]. Moreover, it has been proven that these changes contribute to the accumulation of fragmented and dysfunctional mitochondria in heart failure [215]. MFN2 in the acute phase of ischemic-reperfusion injury causes the binding of the sarcoplasmic reticulum to neighboring mitochondria, increases mitochondrial calcium overload [216], and promotes mitochondrial fragmentation and apoptosis [217]. OPA1 expression is reduced [209,214]. OPA1 mediates mitochondrial inner membrane fusion and maintains mitochondrial cristae integrity and morphology, thus playing a key role in the efficiency of mitochondrial respiration [218].

Additionally, the mitochondrial death and mitophagy marker BNIP3 binds and inhibits OPA1 via its transmembrane domain, promoting mitochondrial fragmentation and apoptosis [219]. Protein kinase A and calcineurin have opposing effects, share many common target proteins such as phospholamban (calcium cycle), troponin I, DRP1 [220,221], and play a key role in the initiation and development of cardiac remodeling during the progression of heart failure [222,223]. These examples and the results of similar studies in the literature indicate that several signaling pathways strictly regulate the post-translational modification of mitochondrial dynamic proteins and modulate mitochondrial morphology, dynamics, and function [224] during the progression of CVDs. This confirms the idea that MD during the follow-up of CVDs is involved in a complex dynamic process of pathologically stimulated development. This certainly requires further study and a deeper understanding of the fundamental ideas behind these processes. It should be noted that the majority of proteins involved in metabolism are mitochondrial proteins and have reduced expression during the progression of CVDs. This process is associated with increased mitochondrial vacuolar degeneration [196].

All of the above data demonstrate the fact that at the stage of the existing cardiovascular continuum, a complex transformation of mitochondrial and cellular signaling is observed that results in changes in metabolism. This is confirmed by the data on significant progressive changes in the oxidation–reduction balance of myocardial mitochondria and their mitochondrial matrix calcium [196]. It has been established that it is the overload of the mitochondrial matrix with calcium and the increased level of ROS observed in the mitochondria of myocardiocytes in CVDs that are the key reasons for further metabolic disorders [44,196]. For example, according to the literature, at the early stages of cardiac remodeling, an increase in the calcium content in the mitochondrial matrix is observed [207], which is regarded as a signaling pathway for further transformations of mitochondrial metabolism [44,196]. There is evidence of the decreased expression of MCU and its subcomplex mitochondrial calcium uptake 1 (Micu1) in heart failure [196]. Recombinant expression of VDAC enhances endoplasmic reticulum–mitochondria contact sites and calcium transfer into mitochondria as well as apoptosis [225,226], etc. Calcium overload-induced mitochondria produce even more ROS. In heart failure, there is a decrease in the activity of the ROS utilization system due to a reduction in the expression and activity of superoxide dismutase 2 [227]. This promotes the further activation of lipid, protein, and nucleic acid peroxidation [34], leading to an increase in metabolic disorders [196] and further progression of the cardiovascular continuum.

Typically, cardiomyocyte metabolism is flexible. Depending on energy requirements and substrate availability, cardiomyocyte metabolism switches between adenosine triphosphate (ATP) formation via fatty acid β-oxidation and glucose metabolism. During periods of prolonged starvation, myocardiocytes utilize ketones [228,229]. In CVDs, cardiomyocyte metabolism undergoes pathological changes due to the development and progression of MD. It has been established that mitochondrial biogenesis and oxidative capacity are preserved in patients with heart failure with normal ejection fraction [209] or are enhanced in pathological compensated hypertrophy [230,231]. In the early stages of CVDs, metabolic remodeling may be adaptive because ATP formation from glycolysis and glucose metabolism is more efficient in terms of oxygen consumption compared with fatty acid metabolism [227]. In experimental models, a decrease in mitochondrial content and oxidative capacity was established during the transition from a compensated state to moderate remodeling and early systolic dysfunction [231]. The transition to heart failure with a reduced ejection fraction is characterized by a decrease in mitochondrial biogenesis and PGC-1α expression [209,230]. Increased acetylation of mitochondrial proteins has been established in animal models and in human heart failure with a reduced ejection fraction [232,233]. This process affects proteins involved in fatty acid beta-oxidation, the tricarboxylic acid cycle, and components of electron chain complexes. This leads to decreased activity of these proteins and disruption of these processes [196]. Progression of CVDs and late stages of heart failure are associated with the reduced expression of genes related to glucose metabolism [234,235,236,237]. At these stages, the amount of ATP produced by glucose metabolism is limited and may be insufficient to meet metabolic needs and increase the load on myocardiocytes. There is also disruption of fatty acid metabolism and beta-oxidation. These metabolic changes lead to the accumulation of harmful lipid products, such as ceramide, in the cytoplasm. This is one of the mechanisms of cardiolipotoxicity due to MD [238]. This contributes to further progression and a decrease in cardiac ejection fraction [196]. As CVDs progress, the contribution of alternative metabolism increases. Increased ketone body oxidation has been shown in animal models and in patients with heart failure [227,237,239,240]. This is associated with elevated circulating ketones in patients with CVDs and is indicative of altered systemic metabolism [237]. Reactome pathways related to branched-chain amino acid catabolism are reduced in compensated hypertrophy and contribute to the transition to heart failure with reduced ejection fraction [241,242]. The accumulation of branched-chain amino acids impairs glucose metabolism and worsens ischemia/reperfusion injury and is reversed by restoration of branched-chain amino acid catabolism [243]. The described changes in the metabolism of cardiomyocytes confirm a significant metabolic transformation in mitochondrial functions at the stage of the existing cardiovascular continuum.

What causes such fundamental changes in the function, metabolism, and morphology of mitochondria at the stage of the existing cardiovascular continuum? Indeed, fundamental changes in intramitochondrial and intracellular signaling must underlie such significant differences in the severity of mitochondrial dysfunction and mitochondrial morphology between the stages of risk factor formation and the stage of the existing cardiovascular continuum. It is well-known that the primary source of all information and the “conductor” of all metabolic processes of cellular activity are deoxyribonucleic acid (DNA) molecules. Therefore, this difference in the pathological change in mitochondria at the stage of existing CVDs should be sought in this direction. The results of the conducted systematic analysis allowed us to conclude that it is the accumulated pathology, in the form of mitochondrial DNA mutations, that is the cause of the ever-increasing pathological transformations of mitochondria during the occurrence and progression of CVDs.

The mitochondrial respiratory chain requires the expression of gene products encoded by nuclear and mitochondrial DNA [244]. Mitochondrial DNA contains 37 genes that encode 13 proteins. These proteins are subunits of respiratory complexes I, III, IV, and V. Complex II is encoded by the nuclear genome. All regulatory factors that control the expression of nuclear and mitochondrial respiratory genes are of nuclear origin [244]. Mitochondrial DNA is located close to the inner mitochondrial membrane, is small in size, and is not protected by histone proteins [245,246]. Therefore, it is a sensitive cellular target for excess ROS, which are formed in the inner mitochondrial membrane. Many different types of oxidative damage to mitochondrial DNA have been described (e.g., from base modifications or sugar adducts to single- and double-strand breaks) [247]. Mutations and damage to mitochondrial DNA are involved in aging, cancer, and neurodegenerative diseases [248] as well as several other pathophysiological conditions [249]. Mitochondrial DNA damage correlates with the degree of atherosclerosis [170] and is associated with atherogenesis [250,251,252]. Accumulation of mitochondrial DNA mutations leads to cellular dysfunction, impaired oxidative phosphorylation, impaired calcium homeostasis, impaired mitochondrial protein metabolism, increased oxidative stress, and increased susceptibility to apoptotic signals [253]. Impaired functioning of respiratory enzymes due to mitochondrial DNA mutations may manifest as electron leakage and pathological ROS production. This forms a vicious circle in which increased oxidative stress leads to further damage to mitochondrial DNA and mitochondrial structures [254]. Thus, during the period of risk factor action and the entire follow-up for CVDs, chronic damage to mitochondrial DNA may occur, leading to the accumulation of mutations and the development of mitochondrial heteroplasmy. Further progression of CVDs is facilitated by the development of more severe disorders of energy-synthetic processes in mitochondria due to an ever-increasing number of mitochondrial DNA mutations. According to the literature, seventeen mutations of the mitochondrial genome have been described in ischemic heart disease, localized in six genes of transport ribonucleic acid (RNA), genes of the 12S ribosomal RNA subunit, and genes of the II and V subunits of NADH dehydrogenase [255]. For example, G1541A and A1555G are nucleotide substitutions located in the 12S ribosomal RNA, causing a decrease in the function of the ribosome [256].

C1624T is localized in the gene of transport RNA-Val. When it is present, cytosine is replaced by uracil in position 25 of transport RNA-Val, as a result of which the secondary structure of the transport RNA changes. This leads to a decrease in the activity of transport RNA-Val. This mutation is associated with hypertrophic cardiomyopathy [256] and is also found in ischemic heart disease [257]. A3243G, C3256T, and A3260G are nucleotide substitutions located in the gene of transfer RNA-Leu (recognition codon UUR), which cause a defect in transfer RNA-Leu and lead to a decrease in its activity. A4269G, A4300G, and A4317G are nucleotide substitutions located in the gene of transfer RNA-Ile, which cause a defect in transfer RNA-Ile and lead to a decrease in its activity. A4833G is a mutation located in the gene of subunit 2 of NADH dehydrogenase, which causes a defect in the protein chain of 2 NADH dehydrogenase and leads to a decrease in the function of the enzyme. C1624T is localized in the gene of transport RNA-Val. When it is present, cytosine is replaced by uracil in position 25 of transport RNA-Val, as a result of which the secondary structure of the transport RNA changes. This leads to a decrease in the activity of transport RNA-Val. This mutation is associated with hypertrophic cardiomyopathy [256] and is also found in ischemic heart disease [257]. A3243G, C3256T, and A3260G are nucleotide substitutions located in the gene of RNA-Leu transfer (recognition codon UUR), which cause a defect in RNA-Leu transfer and lead to a decrease in its activity. A4269G, A4300G, and A4317G are nucleotide substitutions located in the gene of RNA-Ile transfer, which cause a defect in RNA-Ile transfer and lead to a decrease in its activity. A4833G is a mutation located in the gene of subunit 2 of NADH dehydrogenase, which causes a defect in the protein chain of 2 NADH dehydrogenase and leads to a decrease in the function of the enzyme. A8296G and G8363A are nucleotide substitutions located in the gene of transfer RNA-Lys, cause a defect in transfer RNA-Lys, and lead to a decrease in its activity [258]. T9997C is a mutation located in the gene of transfer RNA-Gly, in the presence of which a defect in transfer RNA-Gly is observed, which leads to a decrease in its activity [259]. G12192A is a mutation of the gene of transfer RNA-His, which provokes a defect in transfer RNA-His, leading to a decrease in its activity and causes dilated cardiomyopathy [260]. T12297C and G12315A are nucleotide substitutions located in the Leu transfer RNA gene (CUN recognition codon) that cause a defect in Leu transfer RNA and lead to a decrease in its activity. G13513A is a mutation in the 5th subunit gene of NADH dehydrogenase that causes a defect in the protein chain of the enzyme and leads to a decrease in its function [261]. The G14459A mutation causes a defect in the sixth protein subunit of the mitochondrial respiratory chain enzyme, which leads to dysfunction of NADH dehydrogenase. The association of this mutation with atherosclerotic lesions may be due to the fact that a decrease in the number of normally functioning enzymes in the mitochondria leads to oxidative damage to the intimal cells of human vessels. An association has been established between the degree of heteroplasmy for the 3256T polymorphism in blood leukocytes and atherosclerotic lesions of the carotid arteries [262]. The 3256T polymorphism is localized in the MT-TL1 gene (UUR recognition codon) of the human mitochondrial genome, encoding the tRNA leucine sequence. At the cellular level, this mutation is manifested by a decrease in the number of organelles and a disruption of protein synthesis [263,264], and so on.

An important pathogenetic component of CVD progression at the stage of the existing continuum is the pathological effects caused by dyslipidemia. Oxidized low-density lipoproteins cause damage to mitochondrial structures [265]. The load of macrophages with free cholesterol/formation of foam cells [182] is associated with mitochondrial dysfunction, a decrease in the transmembrane potential of mitochondria, activation of the mitochondrial apoptosis pathway, release of mitochondrial cytochrome C, activation of caspase nine, and an increase in the level of the proapoptotic protein Bax [266,267]. Circulating oxidized low-density lipoproteins are an independent risk factor for the development of atherosclerosis and acute CVDs/complications of the cardiovascular continuum. This is because they cause increased mitochondrial ROS production in endothelial cells, the stimulation of apoptosis [106,268,269], activation of mitochondrial complex II [270], and uncoupling of eNOS and NADPH oxidases [271]. Oxidized low-density lipoproteins induce apoptosis in all cells that participate in atherogenesis. Therefore, the death of endothelial cells, vascular smooth muscle cells, macrophages, and T lymphocytes is stimulated [183,272].

There is evidence that myocardial mitochondria are involved in the pathogenesis of cardiac arrhythmias [273,274,275,276,277] and mitochondrial cardiomyopathies [137]. It should be noted that there is a systemic nature of muscle cell damage under the influence of physical inactivity as a risk factor in the dynamics of the cardiovascular continuum. At the stage of CVDs, mitochondrial dysfunction of cardiomyocytes is combined with mitochondrial dysfunction of skeletal muscles. This plays a fundamental role in reducing the functional capabilities of the bodies of patients with CVDs, manifests itself in a decrease in peak oxygen consumption during physical activity [278,279], is associated with increased oxidative stress, and with the development of cardiac cachexia and concomitant NCDs (for example, diabetes mellitus and obesity) [280]. This is more pronounced in patients at the stage of heart failure with reduced ejection fraction. In these patients, the combination of the mitochondrial dysfunction of skeletal muscles and muscle atrophy is reliably associated with unfavorable outcomes and decreased survival [281]. This indicates the presence of an energy relationship in the human body between the tissues of the muscular system and organs. This indicates the importance of the metabolic role of striated muscles as the main “mitochondrial depot” in metabolism and energy distribution in the human body [130].

Systematic analysis of scientific data has established that at the stage of existing CVDs, MD affects all tissues and organs of the human body to varying degrees. MD is a universal mechanism of NCDs [20,21,282,283]. This explains the fact that at the stage of existing CVDs, there is an increase in the degree of comorbidity. This is quite logical, since the MD of liver cells, endothelium, and vascular intima forms multiple “vicious circles” with components of “classical” pathogenetic mechanisms of atherogenesis, endothelial dysfunction, and cytoenergy deficiency in the tissues of the organs involved [34]. At this stage of the continuum, the primary targets of the influence of MD factors continue to be lipoprotein metabolism in the liver and atherogenesis processes in the vessels. Progression of endothelial dysfunction and atherosclerotic plaques continue, the erosion and rupture of which at the next stage of the continuum will lead to the occurrence of myocardial infarction and acute cerebrovascular accident [34].

At the stage of existing CVDs, significant metabolic changes continue to occur in the liver, which are also caused by the processes of the MD of hepatocytes. Against the background of increased free radical oxidation, the intensity of lipid peroxidation continues to grow. This results in even greater oxidative stress and cellular energy deficiency. Excessive formation of free radicals occurs, which causes ongoing damage to mitochondrial structures and cell membranes. This leads to the death of hepatocytes by necrosis or the rapid initiation of apoptosis. These pathological processes form gradual fibrotic damage and functional disorders of the liver [99,284,285,286,287,288,289]. Meanwhile, activation of free radical processes continues. As a result of excess free fatty acids, which enter the liver in large quantities, conditions are formed for the development of fatty liver disease. Fatty liver disease is a complication of CVDs and other pathologies of NCDs [290]. In this case, the MD in hepatocytes continues to increase. This results in disturbances in tissue respiration and fat oxidation processes, cellular energy deficiency and tissue hypoxia develop, and the hyperactivation of free radical oxidation increases. Further pathological processes are initiated primarily due to tissue hypoxia and the production of excess radicals [100,105,284]. In fatty liver disease, the cell exists in conditions of energy deficiency in the production of natural antioxidant systems. Therefore, progressive liver damage with mitochondrial damage continues, in which bioenergetic hypoxia becomes one of the leading factors influencing the development of fatty liver disease [97,289,290]. At the stage of SSC, it is the hypoxic phenomena associated with adenosine triphosphate deficiency and mitochondrial oxidation disorders that lead to further activation of free radical oxidation. In this case, changes occur in all tissues as a result of the induction of factors that protect the cell from hypoxia. The oxygen-sensitive protein complex plays the most critical role in tissue hypoxia. This hypoxia-inducible factor is activated in the areas of the regulation of oxygen pathways and ensures a rapid response to hypoxia. However, this same factor will be responsible for cell neoplasia [291,292,293,294].

If at the stage of existing CVDs the patient continues to fail to adhere to the principles of a healthy lifestyle, in particular drinking alcohol and smoking, then the above, due to the toxic effect, will increase MD with an even more significant disruption of mitochondrial oxidative phosphorylation, damage to the endoplasmic reticulum, gluconeogenesis, glucose transport with a simultaneous increase in the synthesis of proinflammatory cytokines, and disruption as a result of carbohydrate and lipid metabolism [19,34]. Hypoxia, regardless of the type (tissue or intermittent), will lead to the emergence of conditions characteristic of metabolic syndrome: insulin resistance, disruption of the function of adipose tissue, heart, and endothelial dysfunction [295].

The individual picture of CVDs progression and comorbidity at the stage of the existing cardiovascular continuum can be explained by the varying degree of severity and diversity of manifestations of mitochondrial dysfunction in the tissue structures of organs, resulting from the individual outcome of the organism’s adaptation mechanisms interacting with fundamental atherogenic factors. Logically, this is based on the particular characteristics of the existing stage and degree of the mitochondrial dysfunction of cells and tissues of specific organs and systems involved in the pathological circles of CVDs progression and NCDs. For example, renal mitochondrial dysfunction and hypertension cause glomerular capillary damage and tubulointerstitial inflammation in the kidneys, contribute to a decrease in the glomerular filtration rate due to a reduction in the filtration surface area and the number of nephrons [296,297,298], and increase the risk of renal failure by causing renal hypoxia [299]. Renal medulla hypoxia contributes to the progression of renal damage in chronic kidney disease and hypertension [300]. Mitochondrial dysfunction caused by apoptosis-inducing factor deficiency [301], mitochondrial complex I activity [302], or impaired fatty acid oxidation [303] may contribute to the development of chronic kidney disease. The appearance of mitochondrial DNA in urine is a marker of renal failure and dysfunction in patients with hypertension, which demonstrates mitochondrial damage in renal failure in patients with hypertension [304]. In brain neurons, mitochondrial dysfunction and high levels of mitochondrial ROS lead to cell death and cognitive impairment [305], can provoke neurodegeneration, and increase the risk of stroke [306,307]. Mitochondrial dysfunction, excessive ROS production, and the oxidation of low-density lipids are associated with the pathogenesis of type 2 diabetes mellitus and obesity. ROS can cause inactivation of the signaling pathway between the insulin receptor and the glucose transport system. This leads to insulin resistance [308]. In this case, diabetes mellitus is an additional cause of increased oxidative stress and increased atherogenic effect [309]. Hyperglycemia induces superoxide formation in endothelial cells, and much of this superoxide can be produced by mitochondria [310]. In diabetes mellitus, uncoupling of electron transfer and oxidative phosphorylation occurs. This leads to increased superoxide formation and ineffective ATP synthesis [311]. Increased free fatty acid concentrations with subsequent accumulation of intramyocellular lipids is one of the reasons for the development of further insulin resistance and the death of pancreatic beta cells in diabetes mellitus [312,313]. The systemic effects of mitochondrial dysfunction in humans are supported by the fact that type 2 diabetes mellitus and insulin resistance are associated with decreased mitochondrial oxidative function in skeletal muscle [314].

The progression of ultrastructural changes in the heart characterizes this stage. In the heart, there is disorganization of myofibrils and sarcomeres, damage to gap junctions, changes in nuclear organization, chromatin aggregation, lipid deposition, glycogen aggregation, changes in the endoplasmic reticulum-mitochondria association, dilation of the endoplasmic reticulum, and the presence of autophagosomes and lysosomes [315]. Changes in the shape, content, and distribution of mitochondria accompany this. Mitochondria become swollen and have damage to the cristae. MD is a common sign of adverse remodeling and heart failure [315].

### 2.3. Conceptualization of the Key Aspects of MD at the Stage of Complicated Cardiovascular Continuum

**Figure 4:**
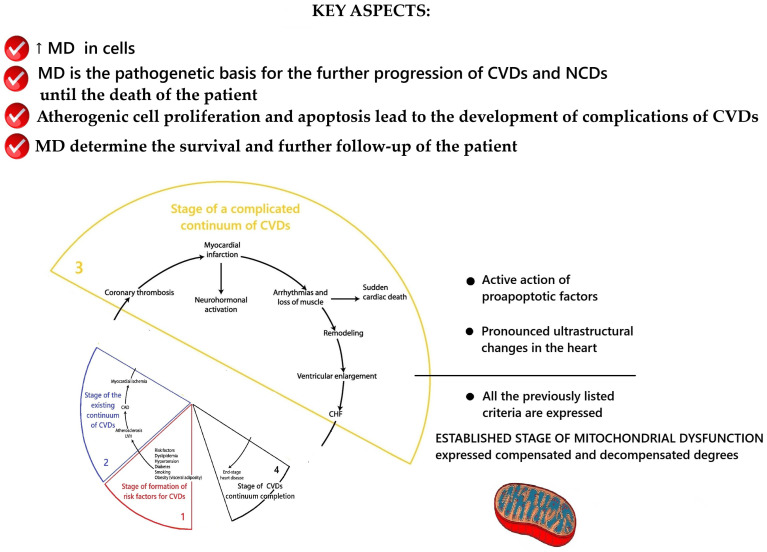
Scheme of the key aspects of MD at the stage of complicated cardiovascular continuum. Fragment [25] used. Note: CAD—coronary artery disease; LVH—left ventricular hypertrophy; CHF—heart failure.

According to V. Dzau and E. Braunwald, the complicated cardiovascular continuum stage occurs when cardiovascular complications appear in the form of acute coronary syndrome, acute myocardial infarction, acute cerebrovascular accident, etc. [24,25,26]. The results of the conceptualization of key aspects of MD at this stage are presented in Figure 4.

At this stage, MD remains involved in the processes of further CVDs progression until the end of the continuum—the death of the patient from CVDs or from another nosology of NCDs. The previously described key mechanisms of mitochondrial dysfunction’s participation in the pathogenesis of CVDs remain. These are the ones that, at certain individual stages of the catamnesis of the disease, lead to a combination of cellular atherogenic proliferation and apoptosis that end in the development of acute cardiovascular events/complications.

Acute coronary syndrome, acute myocardial infarction, and other acute syndromes are based on thrombotic complications of atherosclerosis [183]. These complications arise as a result of rupture of an atherosclerotic plaque [316,317,318]. During atherogenesis, smooth muscle cells play an essential role in stabilizing atherosclerotic plaques. Smooth muscle cell mitochondria participate in the synthesis of a large number of extracellular matrix macromolecules that stabilize atherosclerotic plaques [319,320]. The generation of apoptotic signals by smooth muscle cell mitochondria leads to the death of these cells. This depletes the fibrous capsule and promotes plaque destabilization [321]. Apoptotic endothelial cells [322] and apoptotic smooth muscle cells [323] can promote the onset of coagulation processes due to the action of membrane phosphatidylserine and the loss of anticoagulant membrane components in apoptotic endothelial cells [324]. Thus, for the stage of existing CVDs to pass into the stage of complicated cardiovascular continuum, metabolic disturbances due to MD must reach a critical level of exposure to proapoptotic factors on atherosclerotic plaque cells. According to the results of a systematic analysis of the existing data, the most studied proapoptotic factors are biologically active environmental factors (mechanical action [325,326,327,328,329], oxidative stress [330], radiation [331,332,333,334,335], reactive oxygen or nitrogen species [336,337,338,339], lipids (cholesterol and its oxides) [331,340,341,342,343], viral [344,345,346] and bacterial products [347], and inflammatory cytokines [348,349,350]. As can be seen from this list, the majority of the presented proapoptotic factors arise from the aberrant performance of their functions by the mitochondria of body cells. To date, the key role of mitochondria in the implementation of apoptotic processes has been established [351,352]. Three pathways for the regulation of apoptosis by mitochondria have been discovered [183]: (1) inhibition of mitochondrial respiration due to the production of large amounts of NO in cytokine-stimulated vascular cells [353]; (2) production of cytotoxic reactive oxygen species or changes in cellular oxidation–reduction potential [335,354]; (3) release of proapoptotic molecules, including cytochrome c and apoptosis-inducing factor [355]. All of these allow us to conclude that MD/aberrant mitochondrial function has a critical impact on the development of CVDs, follow-up, and the transition of the cardiovascular continuum to the stage of complications.

A fundamentally important aspect for the life of a patient at the stage of complicated cardiovascular continuum is that it is mitochondrial dysfunction during an acute vascular event/complication that is the critical factor that determines cell death in this case, and in the reperfusion zone. For example, a deficiency in the supply of oxygen and nutrients to cardiomyocytes at the onset of acute myocardial ischemia in patients with acute myocardial infarction leads to critical biochemical and metabolic disturbances in cardiomyocytes, an adverse effect on mitochondrial function, and ATP production [356,357]. During acute myocardial ischemia, cardiomyocyte mitochondria switch to anaerobic glycolysis. This leads to the intracellular accumulation of lactate and protons and reduces the intracellular pH to <7.0. Myocardiocytes remove excess protons in exchange for the influx of sodium ions. Intracellular sodium ion overload occurs, which is associated with a decrease in the activity of Na^+^/K^+^-ATPase due to the depletion of ATP reserves. The Na^+^/Ca^2+^ ion exchanger acts in the opposite mode, trying to remove excess sodium ions. However, this leads to intracellular mitochondrial calcium ion overload [356,357]. The resulting biochemical and metabolic changes, mitochondrial calcium ion overload, oxidative stress, and pH changes are accompanied by the opening of the mitochondrial permeability transition pore [356,357]. The fate of each specific cell during ischemic damage depends on how the morphofunctional state of the mitochondria will allow them to withstand further calcium ion overload, the oxidative surge, and how mitochondrial reenergization occurs. In simple terms, the state of the mitochondria influences the number of cells that will survive the event in the affected area. During an acute vascular event/complication, mitochondria are in a state of increased fragmentation with decreased mitochondrial respiration [358]. In a study [359] on a model of myocardial infarction, it was found that mitochondria become smaller and rounder, their number increases, and the number of autophagosomes increases in the area of the damaged myocardial fiber network. These processes reflect adaptive responses in the heart to the death of cellular structures that have occurred and demonstrate an increase in the processes of the removal of dead components. Mitochondrial fragmentation and dysfunction have been observed in the human myocardium in patients with pressure overload or coronary artery disease with preserved or reduced ejection fraction [209]. The processes of mitophagy and mitochondrial biogenesis are preserved during cardiac surgeries including cardiopulmonary bypass [360,361]. According to some scientists, this reduces damage to mitochondrial DNA [360,361]. Therefore, this means that the state of mitochondria predetermines, to a certain extent, the patient’s survival, the patient’s rehabilitation possibilities after a cardiovascular event/complication, and the further course of their disease after this. Mitochondria predetermine survival and further follow-up at the stage of the complicated cardiovascular continuum.

### 2.4. Conceptualization of the Key Aspects of MD at the Stage of the End of the Cardiovascular Continuum

**Figure 5:**
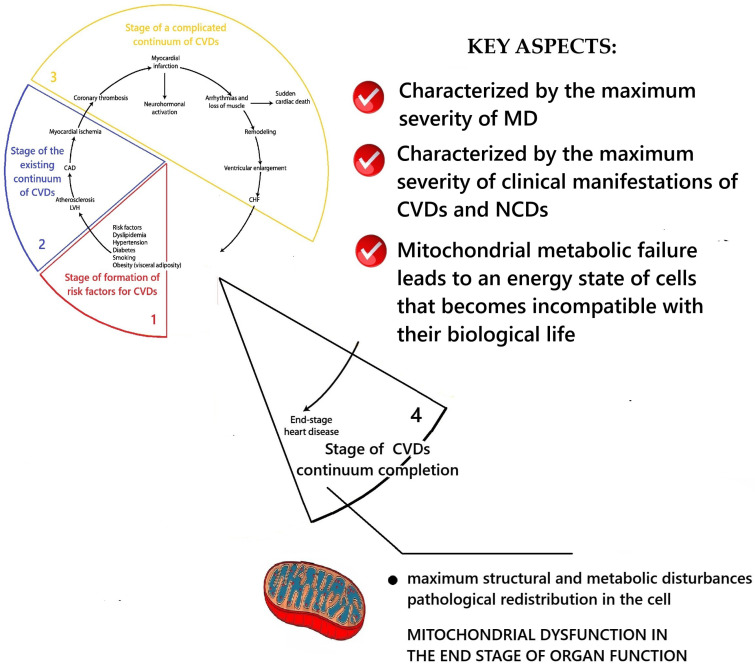
Scheme of the key aspects of MD at the stage of the end of the of the CVDs continuum. Fragment [25] used. Note: CAD—coronary artery disease; LVH—left ventricular hypertrophy; CHF—heart failure.

This is the final stage, at which the CVD follow-up ends due to the patient’s death. The results of the conceptualization of key aspects of MD at this stage are presented in Figure 5.

This stage is characterized by the maximum structural, morphological, and metabolic disturbances of mitochondria as well as their pathological redistribution within cells. The highest degree of mitochondrial dysfunction at this stage leads to metabolic failure and such an energetic state of the myocardium, in which there are gross disturbances/cessation of its function and the death of the patient. Studies of ultrastructural changes in intracellular cardiomyopathy have established extensive changes in mitochondria and autophagic signaling [315]. In the presence of diabetes mellitus, there was damage to mitochondria with destruction of their cristae, disordered arrangement of myofibrils, changes in the nuclear membrane, and increased lipid deposition [362]. A decrease in the area of mitochondria in the cell and the cross-sectional area of collagen fibers, with preservation of the area of the cytoplasm, was established [362]. Several studies have recorded a pathological increase in the size of mitochondria/“megamitochondria” and changes in the glycogen level [363,364,365,366]. Aging is associated with changes in the sarcomeric and mitochondrial structure [315]. A violation of the regulation of gap junctions and the morphology of the intercalated disks of myocardiocytes compared with young hearts [367], a significant decrease in the volume of mitochondria, a reduction in the volume of myofibrils, an increase in the volume of sarcoplasm [368], and the presence of elongated mitochondria/“megamitochondria” were established. These were associated with decreased levels of autophagy protein 9B (Atg9b), nuclear respiratory factor 1 (Nrf1), the ratio between mitochondrial and nuclear DNA, and an increase in the level of mitochondrial-associated p62 [369].

In hypertrophied cardiac tissue, degeneration of cardiac sarcomeres, significant thickening of the z-bands, shortening of the sarcomere, and changes in the basement membrane and tubular structure have been established [370]. In terminal dilated cardiomyopathic cardiac tissue obtained from patients who have undergone heart transplantation, degeneration of cardiac sarcomeres, changes in cytoskeletal proteins, including desmin, have been revealed [371,372]. In cardiac tissue from patients with hypertrophy and valvular disease, focal lysis of myofibrils, loss of thick myofilaments, changes in T-tubules, and expansion of the sarcoplasmic reticulum and mitochondria in these areas have been established [373]. A study of left ventricular endomyocardial biopsies showed decreased density and significant myofiber loss in the hearts of patients with heart failure with a reduced ejection fraction compared with patients with a preserved ejection fraction [374]. A larger population of smaller mitochondria characterized dilated cardiomyopathic hearts compared with ischemic cardiomyopathic hearts. The mitochondrial cell density was 0.3 μm^3^/μm^3^ in the ischemic cardiomyopathic tissue versus ~0.6 in dilated cardiomyopathic heart tissue. This was explained by differential changes in the mitochondrial biogenesis in these CVDs [375]. A study of biopsies from 250 patients with dilated cardiomyopathy confirmed ultrastructural changes in cardiac myofilaments, disorganized sarcomere structure, and mitochondrial and glycogen aggregation [376]. These trends were confirmed in a study of cardiac ultrastructure in endocardial biopsies from patients with chronic heart failure, lysosomal storage diseases, mitochondrial cardiomyopathy, and doxorubicin cardiomyopathy. Depending on the type and stage of the disease, loss of myofibrils, vacuolar degeneration, accumulation of glycogen granules, an increase in the number of autophagic vacuoles, and pathological changes in the size, shape, and number of mitochondria were found [377]. Analysis of autopsy and explanted hearts from patients with decompensated cardiomyopathy and phospholamban mutations revealed the presence of aggresomes containing p62 and LC3, suggesting alterations in autophagy [378]. In a guinea pig model of pressure overload-induced heart failure, mitochondria were fragmented and aggregated, had reduced size, quantitative changes in diameter, and area [379]. In an extensive ultrastructural study of the transition from hypertrophy to heart failure in a mouse model overexpressing myotrophin, mitochondria were found to be swollen, their cristae were disrupted, nuclear membranes were deformed, and myofibrillar and sarcomeric organization at the z-line was disrupted [380]. The presented data highlight the close relationship between the degree of MD and abnormalities in the ultrastructure of the heart during the progression and transition of CVDs to the terminal stage.

## 3. Discussion

The presented results of the system analysis form a new perspective on MD as a dynamic, complex, multifactorial process. The results of the theoretical study demonstrate that quantitative and qualitative changes in the mitochondrial pool of human body cells are the fundamental participants in the pathogenesis of CVDs. Pathological changes in mitochondria during the catamnesis of CVDs and NCDs predetermine the nature and features of the clinical manifestation and course of the disease in each specific patient. Acute cardiovascular complications, as a rule, never occur in the human body at one time against the background of complete health. This is because in order to create local pathological conditions for their occurrence, mitochondria must go through the path of their pathological transformation from functional disorders to systemic metabolic dysfunctions. These mitochondrial transformations during CVDs follow-up are caused by the negative impact of risk factors, accumulated mutations in mitochondrial DNA, and morphological defects of mitochondrial structures on their function.

**Figure 6:**
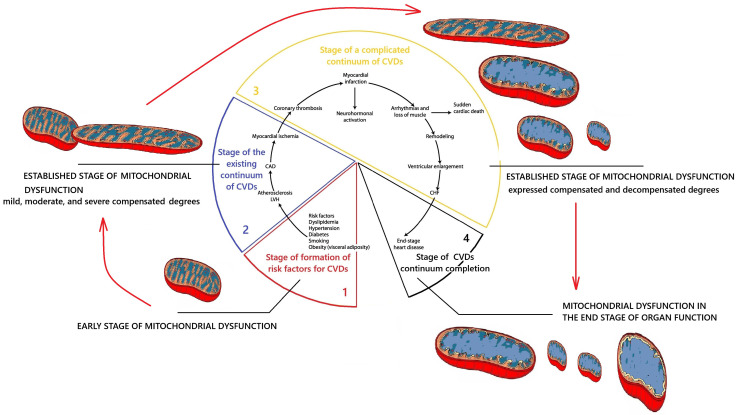
Scheme of the dynamics of MD development during the development of the cardiovascular continuum. Fragment [25] used. Note: CAD—coronary artery disease; LVH—left ventricular hypertrophy; CHF—heart failure.

This review conceptualizes the stages of MD development during the follow-up of CVDs. Based on the data in this review, a proposal is submitted for discussion to the scientific academic community on the need for a differentiated approach to MD depending on the stage of its development and degree. As a working perspective classification, we propose distinguishing the following stages of MD: (1) Initial stage of MD; (2) Formed stage of MD; (3) MD of the terminal stage of organ functioning. Figure 6 demonstrates the dynamics of MD development during the development of the cardiovascular continuum.

The initial stage of MD is characterized by the presence of only functional disorders of mitochondria without their structural and morphological changes, without mutations of mitochondrial DNA, and with minor or moderate disturbances of biogenesis and mitochondrial dynamics. The initial stage of MD is reversible, and mitochondrial function can be restored with the normalization of lifestyle. The formed stage of MD is characterized by the presence of pronounced disturbances of mitochondrial function due to the presence of structural and morphological changes, mutations of mitochondrial DNA, disturbances of biogenesis, and mitochondrial dynamics. The severity of structural and morphological changes, the level of mutational change of mitochondrial DNA, disturbances of biogenesis, and mitochondrial dynamics will predetermine the degree of MD. Therefore, the formed stage of MD can be classified into mild, moderate, severe compensated, and severe decompensated degrees of MD. A severe decompensated degree of MD in the future is transformed into MD of the terminal stage of organ functioning.

From a clinical point of view, it is logical to classify MD by the tissues and organs in which it occurs and is most pronounced.

Of course, further practical development of these ideas requires a scientific definition of specific clinical, laboratory, and morphological criteria for objective gradation of stages and degrees of MD. Since methods for the objective assessment of mitochondrial function suitable for mass routine use in clinical practice have not yet been developed, this remains a purely scientific task for future research. However, the current level of development of knowledge in mitochondriology and its extrapolation to existing knowledge in clinical medicine already allows us to reasonably assert the presence of different stages, degrees, localizations, and other qualitative differences in MD in various organs of the human body in different pathologies.

## 4. Conclusions

MD is a dynamic, complex, multifactorial process characterized by quantitative and qualitative changes in the mitochondrial pool of human body cells during the development of CVDs catamnesis. MD is a fundamental participant in the pathogenesis of CVDs, predetermining the nature and features of the clinical manifestation and course of the disease in each patient. MD has distinctive features at each stage of CVDs catamnesis and can be classified according to this principle. The development of objective methods for assessing the degree of MD and its classification criteria is a promising task for future scientific research.

## Data Availability

No new data were created or analyzed in this study. Data sharing is not applicable to this article.

## Abbreviations

The following abbreviations are used in this manuscript:

MD	Mitochondrial dysfunction
CVDs	Cardiovascular diseases
NCDs	Noncommunicable diseases
CAD	Coronary artery disease
ROS	Reactive oxygen species
ATP	Adenosine triphosphate
CIRS	Cumulative Illness Rating Scale
SCORE	Systematic Coronary Risk Evaluation
DNA	Deoxyribonucleic acid
RNA	Ribonucleic acid
